# Lane changing trajectory planning and tracking control for intelligent vehicle on curved road

**DOI:** 10.1186/s40064-016-2806-0

**Published:** 2016-07-22

**Authors:** Lukun Wang, Xiaoying Zhao, Hao Su, Gongyou Tang

**Affiliations:** Department of Information Engineering, Shandong University of Science and Technology, Taian, China; College of Information Science and Engineering, Ocean University of China, Qingdao, China; College of Foreign Languages, Taishan Medical University, Taian, China

**Keywords:** Lane changing on curved road, Backstepping, B-spline, Sliding-mode control, Trajectory tracking control, Trajectory planning

## Abstract

This paper explores lane changing trajectory planning and tracking control for intelligent vehicle on curved road. A novel arcs trajectory is planned for the desired lane changing trajectory. A kinematic controller and a dynamics controller are designed to implement the trajectory tracking control. Firstly, the kinematic model and dynamics model of intelligent vehicle with non-holonomic constraint are established. Secondly, two constraints of lane changing on curved road in practice (LCCP) are proposed. Thirdly, two arcs with same curvature are constructed for the desired lane changing trajectory. According to the geometrical characteristics of arcs trajectory, equations of desired state can be calculated. Finally, the backstepping method is employed to design a kinematic trajectory tracking controller. Then the sliding-mode dynamics controller is designed to ensure that the motion of the intelligent vehicle can follow the desired velocity generated by kinematic controller. The stability of control system is proved by Lyapunov theory. Computer simulation demonstrates that the desired arcs trajectory and state curves with B-spline optimization can meet the requirements of LCCP constraints and the proposed control schemes can make tracking errors to converge uniformly.

## Background

Intelligent vehicle which can obtain environment information and running state by vehicle sensor is a hot topic of automatic traffic. The current research of intelligent vehicle control includes lane keeping and lane changing which is the process of controlling from one lane to another along the desired trajectory (Rahman et al. 2013; Xu et al. 2012). The lane changing reference trajectory is planned according to vehicle state and road information, and then the controller is designed using onboard sensors to track this virtual trajectory (Hesse and Sattel 2007). There are many kinds of trajectory planning methods, such as trajectory planning based on arcs trajectory which uses the B-spline to optimize running state curve (Elbanhawi et al. 2015). It could overcome the problem of abrupt trajectory curvature change. The trapezoidal acceleration profile resultant trajectory has been known as generating the least possible lateral acceleration on the vehicle (Ren et al. 2011). It adapted to the path planning of straight road. Yang et al. (2012) proposed a near-minimal energy-optimal method to determine the real-time collision-free path for a car-like vehicle. Chen et al. (2012) and Montes et al. (2007) used the Bézier curve to plan trajectory. A continuous smooth trajectory can be obtained by Bézier curve. Shang and Peng (2012) put forward two-lane cellular automaton model which was applied to lane changing with the turn signal effect. Horng et al. (2014) assumed that the vehicle can receive location information about nearby vehicles to perform analysis for lane changing. It simulated the traffic flow with different numbers of lanes. Feng et al. (2015) adopted the polynomial method to plan trajector. Moreover, collision detection was mapped into a parameter space by adopting infinite dynamics circles.

In process of lane changing, the vehicle can get state information according to different types of sensors. Then the kinematic controller and dynamics controller are constructed according to reference velocity and acceleration. Lin and Cook (1997) designed the vehicle lane changing controller which was based on two degrees of freedom dynamics model. The validity of controller was proved by optimal control theory. Naranjo et al. (2008) designed a fuzzy controller for vehicle lane changing under overtaking situation based on high precision GPS positioning system. The lane changing control of vehicle collision avoidance maneuver was explored by Kim et al. (2009). Rastelli and Penas (2015) applied the fuzzy logic technology to the vehicles’ steering control. Hsu and Liu (2008) used lane changing control strategy of vehicle team based on vehicle kinematic model.

The current research on lane changing of intelligent vehicle is mainly based on straight road under the condition that the outside lane curvature and inside lane curvature are both zero, ignoring the effect of longitudinal velocity variation on trajectory. There is less research on lane changing of intelligent vehicle on curved road. This paper aims to study the problem of trajectory planning and tracking control about lane changing on curved road. Assuming that the outside lane curvature and inside lane curvature are greater than zero, the arcs trajectory can be designed. The backstepping technology is used to design the kinematic controller, and the dynamics controller based on sliding-mode method is adopted to extend the kinematic controller. Finally, the effectiveness of the control scheme can be proved by simulation.

## Problem description

### Kinematic model of vehicle

**Fig. 1:**
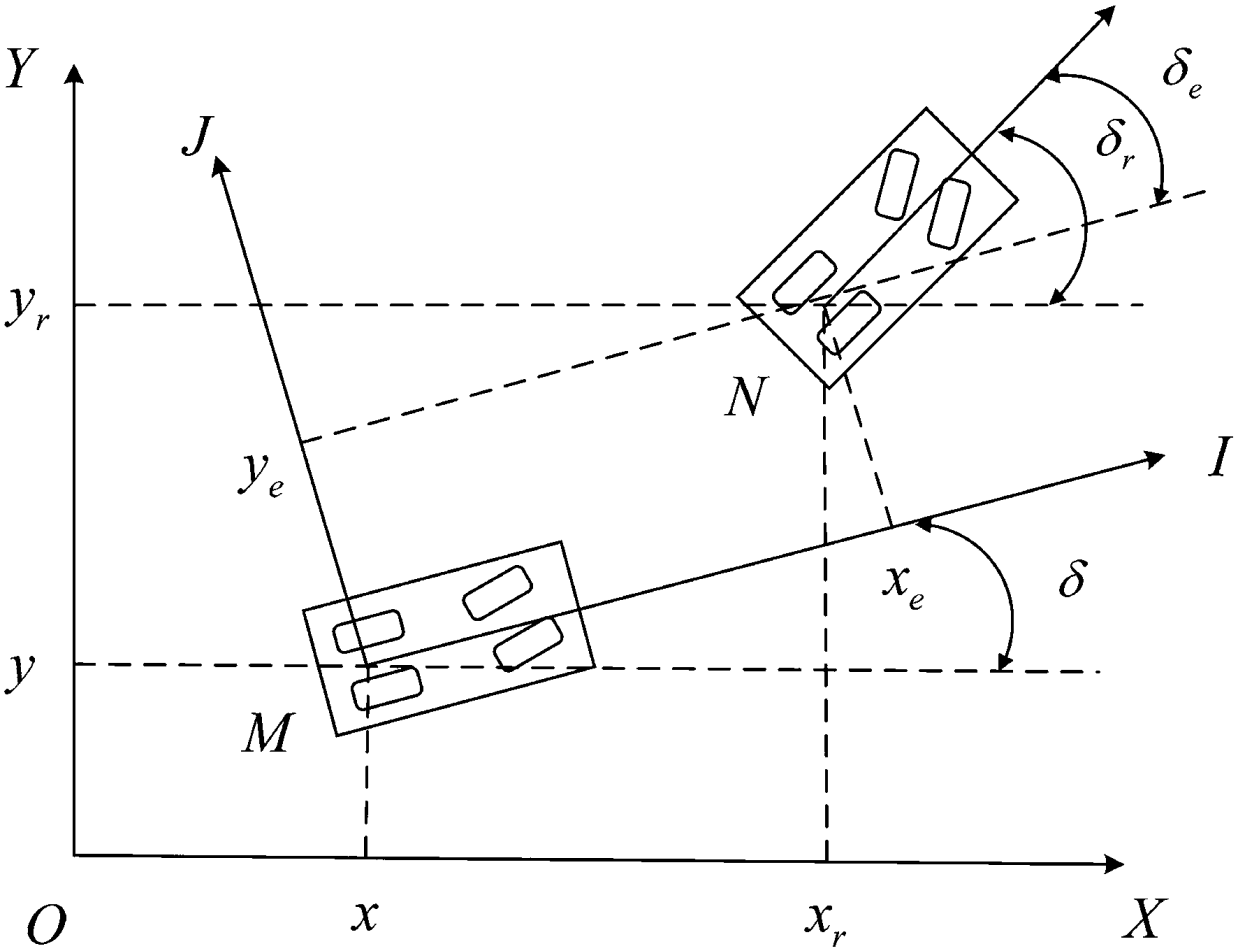
Kinematic model of intelligent vehicle

The intelligent vehicle kinematic model is shown in Fig. 1 in which the world *XOY* coordinate and local *IMJ* coordinate are established. *M* and *N* are the centroid of vehicle.

(*x*, *y*) is the actual position of vehicle in the world coordinate, (*x*_*r*_, *y*_*r*_) is the reference position, *δ* is the angle between *X*-axis and *I*-axis which represents the motion orientation of the vehicle. We use $$ q = \left[ {\begin{array}{*{20}c} x & y & \delta \\ \end{array} } \right]^{T} $$ to represent the actual posture. Suppose that the vehicle is rolling purely without sideslip motion in the world coordinate, the constraint of the vehicle’s motion (Jiang and Nijmeijer 1997) is described as

1 $$ \dot{x}\sin \delta - \dot{y}\cos \delta = 0 $$

Equation (1) can be represented by the form of constraint matrix

2 $$ A\left( q \right)\dot{q} = 0 $$

where $$ A\left( q \right) = \left[ {\begin{array}{*{20}c} {\sin \delta } & { - \cos \delta } & 0 \\ \end{array} } \right] $$ is a vector associated with non-holonomic constraints, $$ \dot{q} = \left[ {\begin{array}{*{20}c} {\dot{x}} & {\dot{y}} & {\dot{\delta }} \\ \end{array} } \right]^{T} $$. On this basis, the kinematic model equation is established (Kanayama et al. 1990)

3 $$ \dot{q} = S\left( q \right)u = \left[ {\begin{array}{*{20}c} {\cos \delta } & 0 \\ {\sin \delta } & 0 \\ 0 & 1 \\ \end{array} } \right]\left[ {\begin{array}{*{20}c} v \\ w \\ \end{array} } \right] $$

where *S*(*q*) is the velocity transformation matrix, $$ u = \left[ {\begin{array}{*{20}c} v & \omega \\ \end{array} } \right]^{T} $$ is the input velocity vector, *v* ∊ *R* and *ω* ∊ *R* denote the linear velocity and angular velocity respectively. Differentiating (3), we can obtain

4 $$ \textit{\"{q}} = S\left( q \right)\dot{u} + \dot{S}\left( q \right)u $$

### Dynamics model of vehicle

The four wheeled intelligent vehicle is a non-holonomic nonlinear system. It is assumed that there is a symmetrical power on both of the left and right sides, the Lagrange equation of three free degrees is established

5 $$ L\left( {q,\dot{q}} \right) = \frac{1}{2}m\dot{x}^{2} + \frac{1}{2}m\dot{y}^{2} + \frac{1}{2}I_{z} \dot{\delta }^{2} $$

where *m* is the mass of vehicle, *I*_*z*_ denotes the moment of inertia. The dynamics of non-holonomic mechanical systems can be described by the differential equation (Campion et al. 1991)

6 $$ \frac{\text{d}}{\text{dt}}\left( {\frac{{\partial L\left( {q,\dot{q}} \right)}}{{\partial \dot{q}}}} \right) - \frac{{\partial L\left( {q,\dot{q}} \right)}}{\partial q} - B\left( q \right)\tau + A^{T} \left( q \right)\lambda = 0 $$

Substituting (5) for (6), the dynamics equation (Fukao et al. 2000) can be defined as

7 $$ M\left( q \right)\textit{\"{q}} + V\left( {q,\dot{q}} \right)\dot{q} = B\left( q \right)\tau - A^{T} \left( q \right)\lambda $$

where *M*(*q*) ∊ *R*^3×3^ is the symmetric inertia matrix, $V\left( {q,\dot{q}} \right) \in R^{3 \times 3}$ is the centripetal and Coriolis matrix associated with the velocity and position, *B*(*q*) ∊ *R*^3×2^ is the input transformation matrix, $$ \tau = \left[ {\begin{array}{*{20}c} {\tau_{l} } & {\tau_{r} } \\ \end{array} } \right]^{T} $$ stands for the torque control inputs vector generated by the right and left driven wheels, *λ* is the Lagrange multiplier, $\dot{q}$ and $\textit{\"{q}}$ represent velocity and acceleration vectors, respectively.

According to (2) and (3), *S*^*T*^(*q*)*A*^*T*^(*q*) = 0 can be derived. If both ends of (7) are pre-multiplied by *S*^*T*^(*q*), the constraint term *A*^*T*^(*p*)*λ* can be eliminated. Meanwhile, substituting (3) and (4) for (7), we can get a dynamics equation in term of internal velocities

8 $$ \bar{M}\left( q \right)\dot{u} + \bar{V}\left( {q,\dot{q}} \right)u = \bar{B}\left( q \right)\tau $$

where $\bar{M}\left( q \right) = S^{T} \left( q \right)M\left( q \right)S\left( q \right)$ is the matrix of system state, $\bar{V}\left( {q,\dot{q}} \right) = S^{T} \left( q \right)\left[ {M\left( q \right)\dot{S}\left( q \right) + V\left( {q,\dot{q}} \right)S\left( q \right)} \right]$ means the matrix of lateral deflection, $\bar{B}\left( q \right) = S^{T} \left( q \right)B\left( q \right)$ denotes the input transformation matrix. In this paper we assume that the friction of surface is neglected and $V\left( {q,\dot{q}} \right) = 0$, the variables in (8) are assumed as

9 $$ M\left( q \right) = \left[ {\begin{array}{*{20}c} m & 0 & 0 \\ 0 & m & 0 \\ 0 & 0 & {I_{z} } \\ \end{array} } \right] $$

10 $$ B\left( q \right) = \frac{1}{r}\left[ {\begin{array}{*{20}c} {\cos \delta } & {\cos \delta } \\ {\sin \delta } & {\sin \delta } \\ b & { - b} \\ \end{array} } \right] $$

where *r* is the wheel’s radius, *b* is half of the distance between two driven wheels.

## Trajectory planning for lane changing

### Lane changing constraint

In recent years, researchers have divided the process of lane changing into two or three segments (Salvucci and Liu 2002; van Winsum et al. 1999). In the process of lane changing, if the starting position of the vehicle is fixed and the finishing position can be arbitrarily setting, it is called lane changing in theory (LCT). With the consideration of the intelligent vehicle dynamics constraints and the characteristics of lane changing on curved road, the constraints of lane changing on curved road in practice (LCCP) are proposed

#### *Constraint 1*

The trajectory of vehicle, velocity and acceleration curve should be continuous smooth curves. An abrupt change of trajectory and state curve is not allowed.

#### *Constraint 2*

The curvature of velocity and acceleration curve should be relatively small in the starting and finishing position. The extremum of curvature will appear in the process of lane changing.

**Fig. 2:**
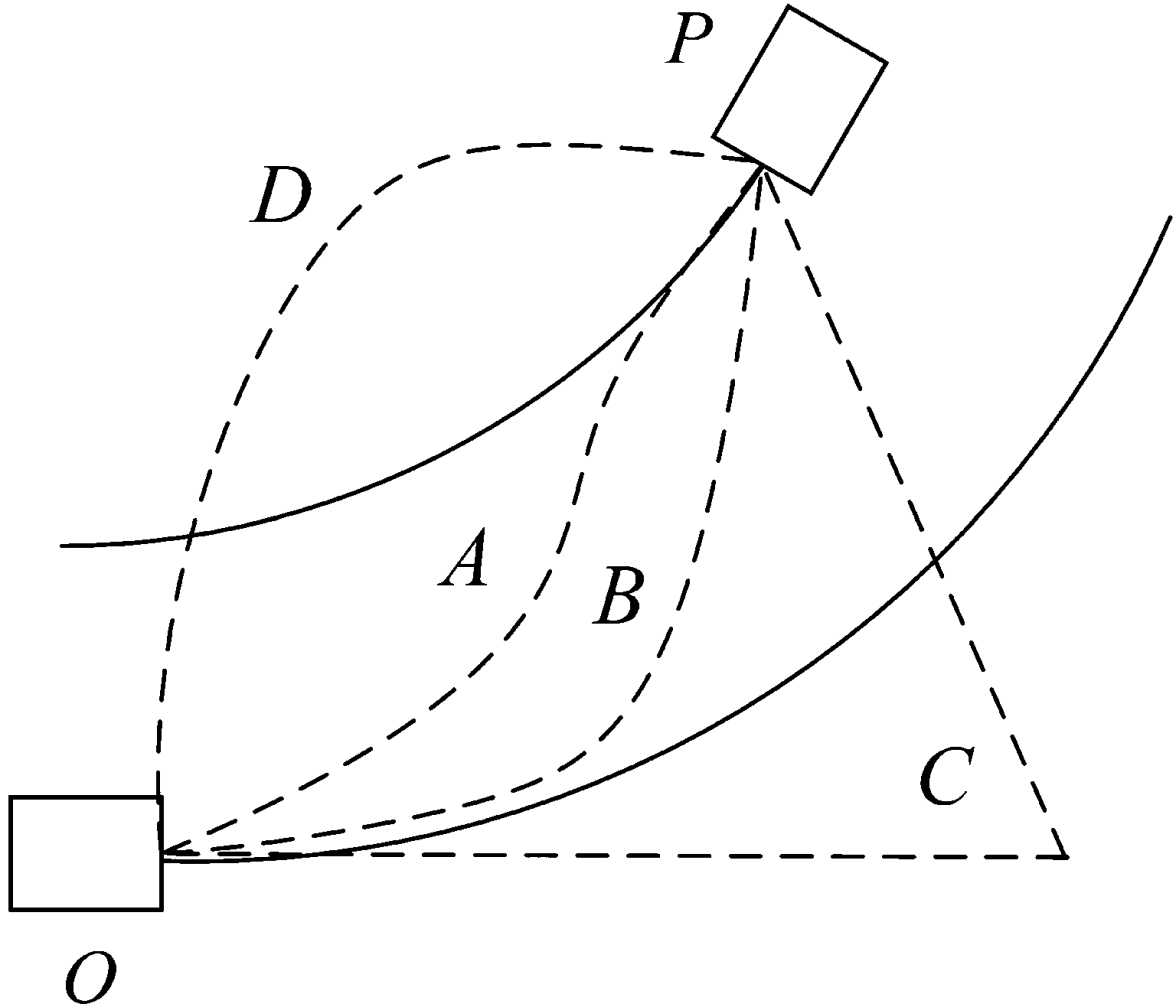
Examples of LCCP and LCT

The examples of LCT and LCCP are shown in Fig. 2. *O* is the starting position while *P* is the finishing position. There are many paths from *O* to *P*. *O* → *C* → *P* is a path which has an abrupt curvature change. It does not meet the LCCP *Constraint 1*. In *O* → *D* → *P*, the extremum of curvature appears in the starting position. It does not meet the LCCP *Constraint 2*. *O* → *A* → *P* and *O* → *B* → *P* are the paths that can be achieved in practice. These two paths can satisfy the LCCP constraints. In addition, there are a great many other paths which can satisfy the LCCP constraints. In this paper, we mainly research the paths composed of two segments arcs which like *O* → *A* → *P*.

### Trajectory planning

**Fig. 3:**
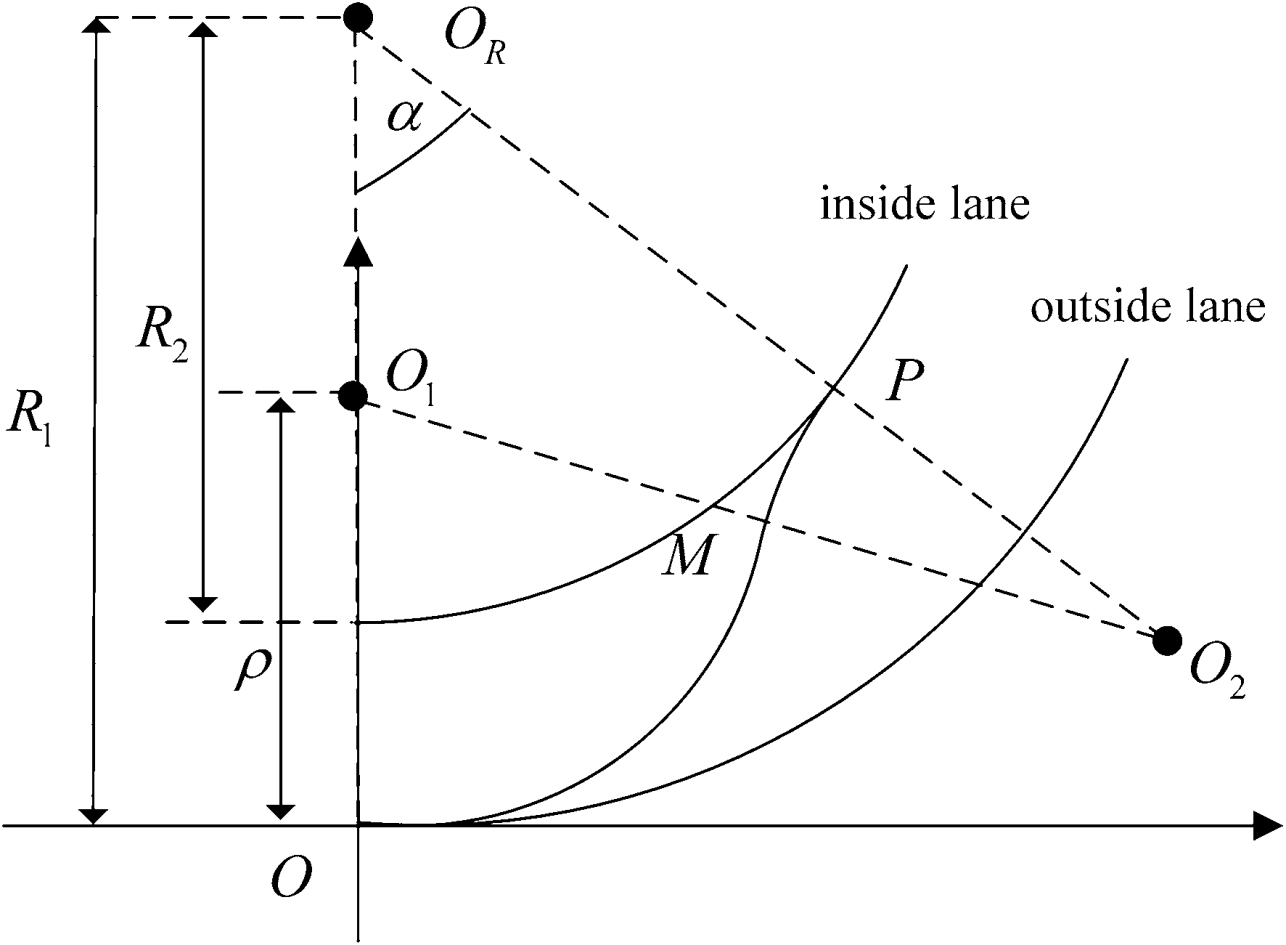
Model of lane changing on curved road

The desired lane changing trajectory is shown in Fig. 3. The position of the vehicle at the starting time is *O* and the finishing position is *P*. The inside and outside lane have the same instantaneous center *O*_*R*_. The curvature radius of outside lane is *R*_1_ and the curvature radius of inside lane is *R*_2_. Assuming that the desired trajectory is composed of two segments arcs which have the equal curvature radius *ρ*. The arcs instantaneous center is *O*_1_ and *O*_2_ respectively. These two arcs are tangent in *M*, *α* is the rotation angel between *OO*_*R*_ and *PO*_*R*_.

**Fig. 4:**
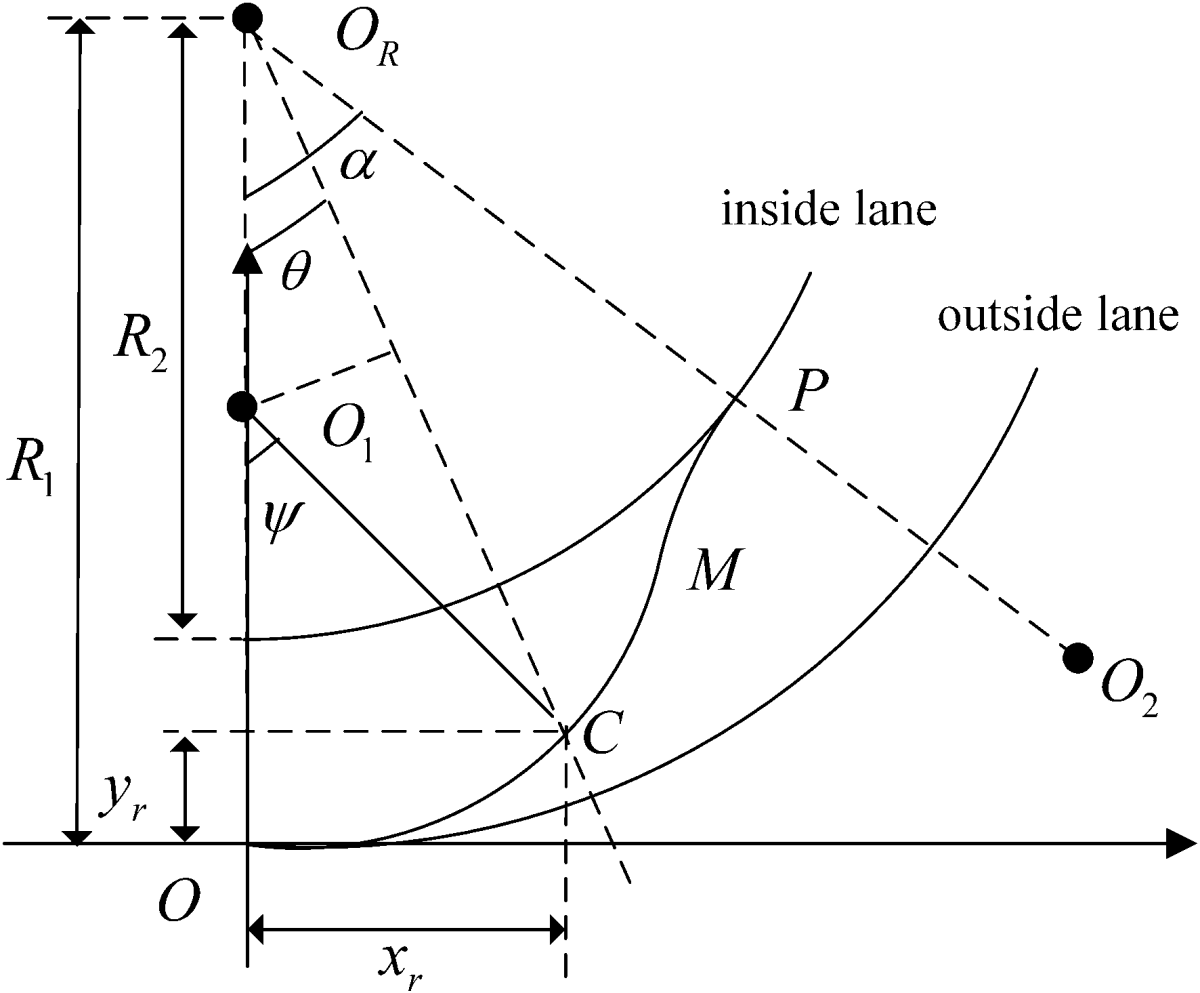
Lane changing on OM segment

Suppose that the centroid of the vehicle moves to the point *C* at time *t* along desired trajectory. If the *C* is in *OM* segment, the trajectory is shown in Fig. 4 where *x*_*r*_(*t*) and *y*_*r*_(*t*) represent the longitudinal and lateral displacement respectively, $\theta$(*t*) is the angel of the vehicle centroid around instantaneous center *O*_*R*_, *ψ*(*t*) denotes the angel of vehicle centroid around instantaneous center *O*_1_, *ψ*(*t*) satisfies

11 $$ \psi \left( t \right) = \theta \left( t \right) + \arcsin \left( {\frac{{\left( {R_{1} - \rho } \right)\sin \theta \left( t \right)}}{\rho }} \right) $$

When the vehicle is in *OM* segment, the desired displacement, velocity, and acceleration can be calculated as

12 $$ \begin{aligned} x_{r} \left( t \right) & = \rho \sin \left[ {\theta \left( t \right) + \arcsin \left( {\left( {R_{1} - \rho } \right)\sin \left( {\theta \left( t \right)} \right)/\rho } \right)} \right] \\ y_{r} \left( t \right) & = \rho - \rho \cos \left[ {\theta \left( t \right) + \arcsin \left( {\left( {R_{1} - \rho } \right)\sin \left( {\theta \left( t \right)} \right)/\rho } \right)} \right] \\ \end{aligned} $$

13 $$ \begin{aligned} \dot{x}_{r} \left( t \right) & = \rho \cos \left[ {\theta \left( t \right) + \arcsin \left( {\left( {R_{1} - \rho } \right)\sin \left( {\theta \left( t \right)} \right)/\rho } \right)} \right] \\ & \quad \left\{ {\dot{\theta }\left( t \right) + \frac{{(R_{1} - \rho )\cos \left( {\theta \left( t \right)} \right)\dot{\theta }\left( t \right)}}{{\rho \sqrt {1 - \left[ {\left( {R_{1} - \rho } \right)\sin \left( {\theta \left( t \right)} \right)/\rho } \right]^{2} } }}} \right\} \\ \dot{y}_{r} \left( t \right) & = \rho \sin \left[ {\theta \left( t \right) + \arcsin \left( {\left( {R_{1} - \rho } \right)\sin \left( {\theta \left( t \right)} \right)/\rho } \right)} \right] \\ & \quad \left\{ {\dot{\theta }\left( t \right) + \frac{{(R_{1} - \rho )\cos \left( {\theta \left( t \right)} \right)\dot{\theta }\left( t \right)}}{{\rho \sqrt {1 - \left[ {\left( {R_{1} - \rho } \right)\sin \left( {\theta \left( t \right)} \right)/\rho } \right]^{2} } }}} \right\} \\ \end{aligned} $$

14 $$ \begin{aligned} \textit{\"{x}}_{r} \left( t \right) & = - \rho \sin \left[ {\theta \left( t \right) + \arcsin \left( {\left( {R_{1} - \rho } \right)\sin \left( {\theta \left( t \right)} \right)/\rho } \right)} \right] \\ & \quad \left\{ {\dot{\theta }\left( t \right) + \frac{{\left[ {(R_{1} - \rho )/\rho } \right]\cos \left( {\theta \left( t \right)} \right)\dot{\theta }\left( t \right)}}{{\sqrt {1 - \left[ {\left( {R_{1} - \rho } \right)\sin \left( {\theta \left( t \right)} \right)/\rho } \right]^{2} } }}} \right\}^{2} \\ & \quad + \rho \cos \left[ {\theta \left( t \right) + \arcsin \left( {\left( {R_{1} - \rho } \right)\sin \left( {\theta \left( t \right)} \right)/\rho } \right)} \right] \\ & \quad \left\{ {\ddot{\theta }\left( t \right) + \frac{{\left[ {(R_{1} - \rho )/\rho } \right]\left[ {\cos \left( {\theta \left( t \right)} \right)\ddot{\theta }\left( t \right) - \sin \left( {\theta \left( t \right)} \right)\dot{\theta }^{2} \left( t \right)} \right]}}{{\sqrt {1 - \left[ {\left( {R_{1} - \rho } \right)\sin \left( {\theta \left( t \right)} \right)/\rho } \right]^{2} } }}} \right. \\ & \left. {\quad + \frac{{\left[ {(R_{1} - \rho )/\rho } \right]^{3} \sin \left( {\theta \left( t \right)} \right)\cos^{2} \left( {\theta \left( t \right)} \right)\dot{\theta }^{2} \left( t \right)}}{{\sqrt[3]{{1 - \left[ {\left( {R_{1} - \rho } \right)\sin \left( {\theta \left( t \right)} \right)/\rho } \right]^{2} }}}}} \right\} \\ \textit{\"{y}}_{r} \left( t \right) & = \rho \cos \left[ {\theta \left( t \right) + \arcsin \left( {\left( {R_{1} - \rho } \right)\sin \left( {\theta \left( t \right)} \right)/\rho } \right)} \right] \\ & \quad \left\{ {\dot{\theta }\left( t \right) + \frac{{\left[ {(R_{1} - \rho )/\rho } \right]\cos \left( {\theta \left( t \right)} \right)\dot{\theta }\left( t \right)}}{{\sqrt {1 - \left[ {\left( {R_{1} - \rho } \right)\sin \left( {\theta \left( t \right)} \right)/\rho } \right]^{2} } }}} \right\}^{2} \\ & \quad + \rho \sin \left[ {\theta \left( t \right) + \arcsin \left( {\left( {R_{1} - \rho } \right)\sin \left( {\theta \left( t \right)} \right)/\rho } \right)} \right] \\ & \quad \left\{ {\ddot{\theta }\left( t \right) + \frac{{\left[ {(R_{1} - \rho )/\rho } \right]\left[ {\cos \left( {\theta \left( t \right)} \right)\ddot{\theta }\left( t \right) - \sin \left( {\theta \left( t \right)} \right)\dot{\theta }^{2} \left( t \right)} \right]}}{{\sqrt {1 - \left[ {\left( {R_{1} - \rho } \right)\sin \left( {\theta \left( t \right)} \right)/\rho } \right]^{2} } }}} \right. \\ & \left. {\quad + \frac{{\left[ {(R_{1} - \rho )/\rho } \right]^{3} \sin \left( {\theta \left( t \right)} \right)\cos^{2} \left( {\theta \left( t \right)} \right)\dot{\theta }^{2} \left( t \right)}}{{\sqrt[3]{{1 - \left[ {\left( {R_{1} - \rho } \right)\sin \left( {\theta \left( t \right)} \right)/\rho } \right]^{2} }}}}} \right\} \\ \end{aligned} $$

In desired trajectory state Eqs. (12)–(14), the *θ*(*t*), $\dot{\theta }\left( t \right)$ and $\ddot{\theta }\left( t \right)$ are unknown functions. In this paper, polynomial method (Feng et al. 2015) is adopt to solve these functions. Assuming that *θ*(*t*) satisfies quantic polynomial

15 $$ \theta \left( t \right) = c_{5} t^{5} + c_{4} t^{4} + c_{3} t^{3} + c_{2} t^{2} + c_{1} t + c_{0} $$

where *c*_*i*_, *i* = 0, …, 5 represent undetermined coefficients. Taking the derivative of *θ*(*t*) with respect to time, the angular velocity and angular acceleration can be obtained

16 $$ \begin{aligned} \dot{\theta }\left( t \right) = 5c_{5} t^{4} + 4c_{4} t^{3} + 3c_{3} t^{2} + 2c_{2} t + c_{1} \hfill \\ \ddot{\theta }\left( t \right) = 20c_{5} t^{3} + 12c_{4} t^{2} + 6c_{3} t + 2c_{2} \hfill \\ \end{aligned} $$

Given *θ*(*t*), $\dot{\theta }\left( t \right)$ and $\ddot{\theta }\left( t \right)$ in starting and finishing position satisfy equations

17 $$ \begin{aligned} \theta \left( {t_{0} } \right) = 0 \hfill \\ \theta \left( T \right) = \alpha \hfill \\ \end{aligned} $$

18 $$ \begin{aligned} \dot{\theta }\left( {t_{0} } \right) = \frac{{\sqrt {v_{x}^{2} \left( {t_{0} } \right) + v_{y}^{2} \left( {t_{0} } \right)} }}{{R_{1} }} \hfill \\ \dot{\theta }\left( T \right) = \frac{{\sqrt {v_{x}^{2} \left( T \right) + v_{y}^{2} \left( T \right)} }}{{R_{2} }} \hfill \\ \end{aligned} $$

19 $$ \begin{aligned} \ddot{\theta }\left( {t_{0} } \right) = \frac{{\sqrt {a_{x}^{2} \left( {t_{0} } \right) + a_{y}^{2} \left( {t_{0} } \right)} }}{{R_{1} }} \hfill \\ \ddot{\theta }\left( T \right) = \frac{{\sqrt {a_{x}^{2} \left( T \right) + a_{y}^{2} \left( T \right)} }}{{R_{2} }} \hfill \\ \end{aligned} $$

where $t_{0} = 0$ is the starting time and *T* is the finishing time, *v*_*x*_(·) and *v*_*y*_(·) are the lateral and longitudinal velocity respectively, *a*_*x*_(·) and *a*_*y*_(·) denote the lateral and longitudinal acceleration respectively. The coefficient vector *C* = [*c*_0_, *c*_1_, *c*_2_, *c*_3_, *c*_4_, *c*_5_]^*T*^can be obtained by solving linear Eqs. (15) and (16) with given conditions (17)–(19). Then *θ*(*t*), $\dot{\theta }\left( t \right)$ and $\ddot{\theta }\left( t \right)$ in (12)–(14) can be gotten.

**Fig. 5:**
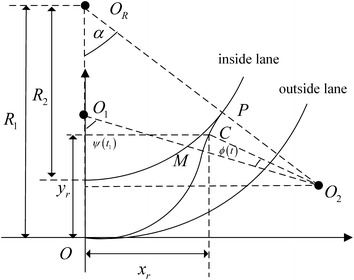
Lane changing on MP segment

If *C* is in *MP* segment, the trajectory is shown in Fig. 5.

The given length between *O*_1_ and *O*_2_ is 2*ρ*, and the length between *O*_*R*_ and *O*_2_ is *R*_2_ + *ρ*. According to this, the coordinates of*O*_2_ can be calculated as ((*R*_2_ + *ρ*)sin *α*, *R*_1_ − (*R*_2_ + *ρ*) cos *α*). Assuming that the vehicle moves to *M* point at the time *t*_1_, $\phi (t)$ denotes the rotation angel of vehicle centroid around instantaneous center *O*_2_ which satisfies

20 $$ \phi \left( t \right) = \int_{{t_{1} }}^{t} {\frac{{\sqrt {v_{x}^{2} \left( t \right) + v_{y}^{2} \left( t \right)} }}{\rho }dt} $$

*ψ*(*t*_1_) and *θ*(*t*_1_) can be calculated as

21 $$ \begin{aligned} \psi \left( {t_{1} } \right) & = \arcsin \left[ {\frac{{\left( {R_{2} + \rho } \right)\sin \alpha }}{2\rho }} \right] \\ \theta \left( {t_{1} } \right) & = c_{5} t_{1}^{5} + c_{4} t_{1}^{4} + c_{3} t_{1}^{3} + c_{2} t_{1}^{2} + c_{1} t_{1} + c_{0} \\ &  = \arctan \left[ {\frac{{x_{r} \left( {t_{1} } \right)}}{{R_{1} - y_{r} \left( {t_{1} } \right)}}} \right] \\ \end{aligned} $$

where *c*_*i*_, *i* = 0, …, 5 have been solved in (15) and (16), $x_{r} \left( {t_{1} } \right) = \rho \sin \left[ {\psi \left( {t_{1} } \right)} \right]$, $y_{r} \left( {t_{1} } \right) = \rho - \rho \cos \left[ {\psi \left( {t_{1} } \right)} \right]$. The given arc length *L* from *O* to *M* is *ψ*(*t*_1_) × *ρ* where *ψ*(*t*_1_) can be obtained by (21), and *L* also satisfies

22 $$ L = \int_{{t_{0} }}^{{t_{1} }} {t\sqrt {v_{x}^{2} \left( t \right) + v_{y}^{2} \left( t \right)} } dt $$

Furthermore, *t*_1_ can be solved by (12), (21) and (22). When the vehicle is in *MP* segment, the desired displacement, velocity, and acceleration can be calculated as

23 $$ \begin{aligned} x_{r} \left( t \right) & = \left( {R_{2} + \rho } \right)\sin \alpha - \rho \cos \left( {\frac{\pi }{2}} \right.\left. { - \arcsin \left[ {\frac{{\left( {R_{2} + \rho } \right)\sin \alpha }}{2\rho }} \right] + \int_{{t_{1} }}^{t} {\frac{{\sqrt {v_{x}^{2} \left( t \right) + v_{y}^{2} \left( t \right)} }}{\rho }} dt} \right) \\ y_{r} \left( t \right) & = R_{1} - \left( {R_{2} + \rho } \right)\cos \alpha + \rho \sin \left( {\frac{\pi }{2}} \right.\left. { - \arcsin \left[ {\frac{{\left( {R_{2} + \rho } \right)\sin \alpha }}{2\rho }} \right] + \int_{{t_{1} }}^{t} {\frac{{\sqrt {v_{x}^{2} \left( t \right) + v_{y}^{2} \left( t \right)} }}{\rho }dt} } \right) \\ \end{aligned} $$

24 $$ \begin{aligned} \dot{x}_{r} \left( t \right) = \sqrt {v_{x}^{2} \left( t \right) + v_{y}^{2} \left( t \right)} \sin \left( {\frac{\pi }{2} - \arcsin \left[ {\frac{{\left( {R_{2} + \rho } \right)\sin \alpha }}{2\rho }} \right]} \right.\left. { + \int_{{t_{1} }}^{t} {\frac{{\sqrt {v_{x}^{2} \left( t \right) + v_{y}^{2} \left( t \right)} }}{\rho }dt} } \right) \hfill \\ \dot{y}_{r} \left( t \right) = \sqrt {v_{x}^{2} \left( t \right) + v_{y}^{2} \left( t \right)} \sin \left( {\frac{\pi }{2}} \right. - \arcsin \left[ {\frac{{\left( {R_{2} + \rho } \right)\sin \alpha }}{2\rho }} \right]\left. { + \int_{{t_{1} }}^{t} {\frac{{\sqrt {v_{x}^{2} \left( t \right) + v_{y}^{2} \left( t \right)} }}{\rho }dt} } \right) \hfill \\ \end{aligned} $$

25 $$ \begin{aligned} \textit{\"{x}}_{r} \left( t \right) & = \left[ {\frac{{v_{x} \left( t \right)\dot{v}_{x} \left( t \right) + v_{y} \left( t \right)\dot{v}_{y} \left( t \right)}}{{\sqrt {v_{x}^{2} \left( t \right) + v_{y}^{2} \left( t \right)} }}} \right]\sin \left( {\frac{\pi }{2}} \right. - \arcsin \left[ {\frac{{\left( {R_{2} + \rho } \right)\sin \alpha }}{2\rho }} \right]\left. { + \int_{{t_{1} }}^{t} {\frac{{\sqrt {v_{x}^{2} \left( t \right) + v_{y}^{2} \left( t \right)} }}{\rho }dt} } \right) \\ & \quad + \frac{{v_{x}^{2} \left( t \right) + v_{y}^{2} \left( t \right)}}{\rho }\cos \left( {\frac{\pi }{2} - \arcsin \left[ {\frac{{\left( {R_{2} + \rho } \right)\sin \alpha }}{2\rho }} \right]} \right.\left. { + \int_{{t_{1} }}^{t} {\frac{{\sqrt {v_{x}^{2} \left( t \right) + v_{y}^{2} \left( t \right)} }}{\rho }dt} } \right) \\ \textit{\"{y}}_{r} \left( t \right) & = \left[ {\frac{{v_{x} \left( t \right)\dot{v}_{x} \left( t \right) + v_{y} \left( t \right)\dot{v}_{y} \left( t \right)}}{{\sqrt {v_{x}^{2} \left( t \right) + v_{y}^{2} \left( t \right)} }}} \right]\cos \left( {\frac{\pi }{2}} \right.\left. { - \arcsin \left[ {\frac{{\left( {R_{2} + \rho } \right)\sin \alpha }}{2\rho }} \right] + \int_{{t_{1} }}^{t} {\frac{{\sqrt {v_{x}^{2} \left( t \right) + v_{y}^{2} \left( t \right)} }}{\rho }dt} } \right) \\ & \quad - \frac{{v_{x}^{2} \left( t \right) + v_{y}^{2} \left( t \right)}}{\rho }\sin \left( {\frac{\pi }{2} - \arcsin \left[ {\frac{{\left( {R_{2} + \rho } \right)\sin \alpha }}{2\rho }} \right]} \right.\left. { + \int_{{t_{1} }}^{t} {\frac{{\sqrt {v_{x}^{2} \left( t \right) + v_{y}^{2} \left( t \right)} }}{\rho }dt} } \right) \\ \end{aligned} $$

In (23)–(25), *v*_*x*_(*t*) and *v*_*y*_(*t*) are unknown functions. We also use the polynomial method to solve these functions. Assuming that *v*_*x*_(*t*) and *v*_*y*_(*t*) satisfies cubic polynomial

26 $$ \begin{aligned} v_{x} \left( t \right) & = a_{3} t^{3} + a_{2} t^{2} + a_{1} t + a_{0} \\ v_{y} \left( t \right) & = b_{3} t^{3} + b_{2} t^{2} + b_{1} t + b_{0} \\ \end{aligned} $$

where *a*_*i*_, *i* = 0, …, 3 and *b*_*i*_, *i* = 0, …, 3 represent the undetermined coefficients. Taking the derivatives of polynomials in (26) with respect to time, one can obtain

27 $$ \begin{aligned} \dot{v}_{x} \left( t \right) & = 3a_{3} t^{2} + 2a_{2} t + a_{1} \\ \dot{v}_{y} \left( t \right) & = 3b_{3} t^{2} + 2b_{2} t + b_{1} \\ \end{aligned} $$

Given *v*_*x*_(*t*_0_), *v*_*x*_(*T*), $\dot{v}_{x} \left( {t_{0} } \right)$, $\dot{v}_{x} \left( T \right)$, *v*_*y*_(*t*_0_), *v*_*y*_(*T*), $\dot{v}_{y} \left( {t_{0} } \right)$, $\dot{v}_{y} \left( T \right)$, $t_{0}  = 0$ and *T*, the coefficient vector *A* = [*a*_0_, *a*_1_, *a*_2_, *a*_3_]^T^ and *B* = [*b*_0_, *b*_1_, *b*_2_, *b*_3_]^T^ can be obtained by solving polynomials (26) and (27). Then *v*_*x*_(*t*) and *v*_*y*_(*t*) in (23)–(25) can be gotten.

With the requirement of vehicle trajectory tracking, the reference yaw angle and yaw angular velocity which are adopted for the input of kinematic trajectory tracking controller can be determined as

28 $$ \delta_{r} \left( t \right) = \arctan \left[ {\frac{{\dot{y}_{r} \left( t \right)}}{{\dot{x}_{r} \left( t \right)}}} \right] $$

29 $$ \dot{\delta }_{r} \left( t \right) = \frac{{\textit{\"{y}}_{r} \left( t \right)\dot{x}_{r} \left( t \right) - \dot{y}_{r} \left( t \right)\textit{\"{x}}_{r} \left( t \right)}}{{\dot{x}_{r}^{2} \left( t \right) + \dot{y}_{r}^{2} \left( t \right)}} $$

## Design of trajectory tracking controller

### Design of kinematic controller

Assuming that the reference posture of vehicle is $$ q_{r} = \left[ {\begin{array}{*{20}c} {x_{r} } & {y_{r} } & {\delta_{r} } \\ \end{array} } \right]^{T} $$ and the actual posture of vehicle is $$ q = \left[ {\begin{array}{*{20}c} x & y & \delta \\ \end{array} } \right]^{T} $$. The posture tracking error (Kanayama et al. 1990) can be described as

30 $$ \left[ \begin{aligned} x_{e} \hfill \\ y_{e} \hfill \\ \delta_{e} \hfill \\ \end{aligned} \right] = \left[ {\begin{array}{*{20}c} {\cos \delta } & {\sin \delta } & 0 \\ { - \sin \delta } & {\cos \delta } & 0 \\ 0 & 0 & 1 \\ \end{array} } \right]\left[ {\begin{array}{*{20}c} {x_{r} - x} \\ {y_{r} - y} \\ {\delta_{r} - \delta } \\ \end{array} } \right] $$

Differentiating (30), a differential equation tracking error can be obtained

31 $$ \left[ \begin{aligned} \dot{x}_{e} \hfill \\ \dot{y}_{e} \hfill \\ \dot{\delta }_{e} \hfill \\ \end{aligned} \right] = \left[ {\begin{array}{*{20}c} {\omega_{c} y_{e} - v_{c} + v_{r} \cos \delta_{e} } \\ { - \omega_{c} x_{e} + v_{r} \sin \delta_{e} } \\ {\omega_{r} - \omega_{c} } \\ \end{array} } \right] $$

The problem of trajectory tracking is to design the control input $$ u_{c} = \left[ {\begin{array}{*{20}c} {v_{c} } & {\omega_{c} } \\ \end{array} } \right]^{T} $$ to make sure that the posture tracking error $$ q_{e} = \left[ {\begin{array}{*{20}c} {x_{e} } & {y_{e} } & {\delta_{e} } \\ \end{array} } \right]^{T} $$ converge to zero, i.e. lim_*t*→∞_[|*x*_*e*_(*t*)| + |*y*_*e*_(*t*)| + |*δ*_*e*_(*t*)|] = 0.

The nonlinear system can be decomposed into several subsystems. The Lyapunov function and virtual control variables for each subsystem can be defined and the vehicle kinematic controller is designed which makes the tracking errors to converge uniformly.

#### **Theorem 1**

*Posture tracking error* (30) *will asymptotically converge to zero vectors if kinematic trajectory tracking controller*

32 $$ u_{c} = \left[ {\begin{array}{*{20}c} {v_{c} } \\ {\omega_{c} } \\ \end{array} } \right] = \left[ {\begin{array}{*{20}c} \begin{aligned} v_{r} \cos \delta_{e} + y_{e} \omega_{c} - k_{1} \lambda \frac{{1 + e^{{ - \omega_{c} }} + \omega_{c} e^{{ - \omega_{c} }} }}{{\left( {1 + e^{{ - \omega_{c} }} } \right)^{2} }}\dot{\omega }_{c} y_{e} + k_{1} \frac{{\lambda \omega_{c}^{2} }}{{1 + e^{{ - \omega_{c} }} }}x_{e} \hfill \\ - k_{1} v_{r} \frac{{\lambda \omega_{c} }}{{1 + e^{{ - \omega_{c} }} }}\sin \delta_{e} + k_{2} x_{e} - k_{1} k_{2} \frac{{\lambda \omega_{c} }}{{1 + e^{{ - \omega_{c} }} }}y_{e} \hfill \\ \end{aligned} \\ {\omega_{r} + 2k_{3} v_{r} y_{e} \cos \frac{{\delta_{e} }}{2} + k_{4} \sin \frac{{\delta_{e} }}{2}} \\ \end{array} } \right] $$

*is applied where λ, k*_*1*_*, k*_*2*_*, k*_*3*_*and k*_*4*_*are positive constants,*$\dot{\omega }_{c}$*and*$\dot{\delta }_{e}$*are defined as*

33 $$ \begin{aligned} \dot{\omega }_{c} & = \dot{\omega }_{r} + 2k_{3} \left( {v_{r} \dot{y}_{e} + \dot{v}_{r} y_{e} } \right)\cos \frac{{\delta_{e} }}{2} \\ & \quad - k_{3} v_{r} y_{e} \dot{\delta }_{e} \sin \frac{{\delta_{e} }}{2} + \frac{1}{2}k_{4} \dot{\delta }_{e} \cos \frac{{\delta_{e} }}{2} \\ \end{aligned} $$

34 $$ \dot{\delta }_{e} = 2k_{3} v_{r} y_{e} \cos \frac{{\delta_{e} }}{2} + k_{4} \sin \frac{{\delta_{e} }}{2} $$

#### *Proof*

Since the variable *y*_*e*_ in (30) is indirectly controlled, a virtual error $\bar{x}_{e}$ is defined as

35 $$ \bar{x}_{e} = x_{e} - k_{1} \frac{{\lambda \omega_{c} }}{{1 + e^{{ - \omega_{c} }} }}y_{e} $$

where *k*_*1*_ *∊* *R*^+^*, λ* *∊* *R*^+^ and $k_{1} \frac{{\lambda \omega_{c} }}{{1 + e^{{ - \omega_{c} }} }}y_{e}$ is the virtual feedbacks. Differentiating (35), the following equation can be obtained

36 $$ \dot{\bar{x}}_{e} = \dot{x}_{e} - k_{1} \lambda \frac{{1 + e^{{ - \omega_{c} }} + \omega_{c} e^{{ - \omega_{c} }} }}{{\left( {1 + e^{{ - \omega_{c} }} } \right)^{2} }}\dot{\omega }_{c} y_{e} - k_{1} \frac{{\lambda \omega_{c} }}{{1 + e^{{ - \omega_{c} }} }}\dot{y}_{e} $$

where $\dot{y}_{e}  =  - \omega_{c} x_{e} + v_{r} \sin \delta_{e}$ and $\dot{x}_{e}  = \omega_{c} y_{e} - v_{c} + v_{r} \cos \delta_{e}$. Let Lyapunov function is defined as

37 $$ V_{1} = \frac{1}{2}\bar{x}_{e}^{2} + \frac{1}{2}y_{e}^{2} + \frac{2}{{k_{3} }}\left( {1 - \cos \frac{{\delta_{e} }}{2}} \right) $$

$$\hfill\square$$

Substituting (35), (36) for differentiation of (37), we obtain

38 $$ \begin{aligned} \dot{V}_{1} &= \bar{x}_{e} \dot{\bar{x}}_{e} + y_{e} \dot{y}_{e} + \frac{{\dot{\delta }_{e} }}{{k_{3} }}\sin \frac{{\delta_{e} }}{2} \\ & = \bar{x}_{e} \left[ {\dot{x}_{e} - k_{1} \lambda \frac{{1 + e^{{ - \omega_{c} }} + \omega_{c} e^{{ - \omega_{c} }} }}{{\left( {1 + e^{{ - \omega_{c} }} } \right)^{2} }}\dot{\omega }_{c} y_{e} - k_{1} \frac{{\lambda \omega_{c} }}{{1 + e^{{ - \omega_{c} }} }}\dot{y}_{e} } \right] + y_{e} \left( { - \omega_{c} x_{e} + v_{r} \sin \delta_{e} } \right) + \frac{{\omega_{r} - \omega_{c} }}{{k_{3} }}\sin \frac{{\delta_{e} }}{2} \\ & = \bar{x}_{e} \left[ {\left( {v_{r} \cos \delta_{e} - v_{c} + y_{e} \omega_{c} } \right) - k_{1} \lambda \frac{{1 + e^{{ - \omega_{c} }} + \omega_{c} e^{{ - \omega_{c} }} }}{{\left( {1 + e^{{ - \omega_{c} }} } \right)^{2} }}\dot{\omega }_{c} y_{e} \left. { - k_{1} \frac{{\lambda \omega_{c} }}{{1 + e^{{ - \omega_{c} }} }}\left( { - \omega_{c} x_{e} + v_{r} \sin \delta_{e} } \right)} \right]} \right. \\ & \quad + y_{e} \left( { - \omega_{c} \bar{x}_{e} - k_{1} \frac{{\lambda \omega_{c}^{2} }}{{1 + e^{{ - \omega_{c} }} }}y_{e} + v_{r} \sin \delta_{e} } \right) + \frac{{\omega_{r} - \omega_{c} }}{{k_{3} }}\sin \frac{{\delta_{e} }}{2} \\ & = \bar{x}_{e} \left[ {\left( {v_{r} \cos \delta_{e} - v_{c} + y_{e} \omega_{c} } \right) - k_{1} \lambda \frac{{1 + e^{{ - \omega_{c} }} + \omega_{c} e^{{ - \omega_{c} }} }}{{\left( {1 + e^{{ - \omega_{c} }} } \right)^{2} }}\dot{\omega }_{c} y_{e} \left. { - k_{1} \frac{{\lambda \omega_{c} }}{{1 + e^{{ - \omega_{c} }} }}\left( { - \omega_{c} x_{e} + v_{r} \sin \delta_{e} } \right)} \right]} \right. \\ & \quad - k_{1} \frac{{\lambda \omega_{c}^{2} }}{{1 + e^{{ - \omega_{c} }} }}y_{e}^{2} + \frac{1}{{k_{3} }}\sin \frac{{\delta_{e} }}{2}\left[ {\left( {\omega_{r} - \omega_{c} } \right) + 2k_{3} v_{r} y_{e} \cos \frac{{\delta_{e} }}{2}} \right] \\ \end{aligned} $$

Substituting (32) for (38), we obtain

39 $$ \begin{aligned} \dot{V}_{1} &= \bar{x}_{e} \left( { - k_{2} x_{e} + k_{1} k_{2} \frac{{\lambda \omega_{c} }}{{1 + e^{{ - \omega_{c} }} }}y_{e} } \right) - k_{1} \frac{{\lambda \omega_{c}^{2} }}{{1 + e^{{ - \omega_{c} }} }}y_{e}^{2} - \frac{{k_{4} }}{{k_{3} }}\sin^{2} \frac{{\delta_{e} }}{2} \hfill \\  &= - k_{2} \bar{x}_{e}^{2} - k_{1} \frac{{\lambda \omega_{c}^{2} }}{{1 + e^{{ - \omega_{c} }} }}y_{e}^{2} - \frac{{k_{4} }}{{k_{3} }}\sin^{2} \frac{{\delta_{e} }}{2} \hfill \\ \end{aligned} $$

It is obvious that the Lyapunov function $\dot{V}_{1} \le 0$. *V*_1_ is a continuous differentiable uniformly function, and $\dot{V}_{1}$ is a negative semi-definite continuous uniformly function. According to the Baralat’s lemma (Brockett 1983), it will know that $\dot{V}_{1} \to 0$ when *t* → ∞ such that $\bar{x}_{e}^{2}$, $\frac{{\lambda \omega_{c}^{2} }}{{1 + e^{{ - \omega_{c} }} }}y_{e}^{2}$ and $\sin^{2} \frac{{\delta_{e} }}{2}$ converge to a zero vector. Considering that the *ω*_*c*_ does not converge to zero, it means that $\mathop {\lim }\nolimits_{t \to \infty } \frac{{\lambda \omega_{c}^{2} }}{{1 + e^{{ - \omega_{c} }} }}y_{e}^{2} = 0$ when lim_*t*→∞_*y*_*e*_ = 0. Because $\mathop {\lim }\nolimits_{t \to \infty } \bar{x}_{e}^{2} = 0$ and lim_*t*→∞_*y*_*e*_ = 0, it is obvious that $\mathop {\lim }\nolimits_{t \to \infty } x_{e} = k_{1} \frac{{\lambda \omega_{c} }}{{1 + e^{{ - \omega_{c} }} }}y_{e}$ and lim_*t*→∞_*x*_*e*_ = 0. Taking into account the fact that *δ*_*e*_ is the practical angle error, the periodicity can be neglected. Hence it can be known that *δ*_*e*_ ∊ [0, 2*π*). Because $\mathop {\lim }\nolimits_{t \to \infty } \sin^{2} \frac{{\delta_{e} }}{2} = 0$ and *δ*_*e*_ ∊ [0, 2*π*), so lim_*t*→∞_*δ*_*e*_ = 0. Under the action of controller (32), the posture tracking error $$ q_{e} = \left[ {\begin{array}{*{20}c} {x_{e} } & {y_{e} } & {\delta_{e} } \\ \end{array} } \right]^{T} $$ is uniformly bounded and lim_*t*→∞_[|*x*_*e*_(*t*)| + |*y*_*e*_(*t*)| + |*δ*_*e*_(*t*)|] = 0, system is asymptotically stable.

### Design of dynamics controller

The sliding-mode control (Utkin 1977) is a kind of nonlinear control method which can overcome the external disturbance. The state-feedback control law can switch from one continuous structure to another based on the current position in the state space. Because of its good robustness, the sliding-mode control technique is suit to track the trajectory of intelligent vehicle.

In this paper, the sliding-mode dynamics controller is designed to make the actual velocities of intelligent vehicle converge to the control velocities generated from the kinematic controller. The error between the actual velocity and kinematic control input is introduced

40 $$ u_{e} = u - u_{c} $$

where *u* = [*v*, *ω*]^*T*^ represents the actual velocity, *u*_*c*_ = [*v*_*c*_, *ω*_*c*_]^*T*^ is the control input associated with the kinematic controller. We select PI-type sliding surface *s*(*t*) as

41 $$ s\left( t \right) = \left[ {\begin{array}{*{20}c} {s_{1} \left( t \right)} \\ {s_{2} \left( t \right)} \\ \end{array} } \right] = u_{e} + \beta \int_{0}^{t} {u_{e} dt} $$

where *β* > 0 is the sliding-surface integral parameter. Taking the derivative of *s*(*t*) with respect to time, one can be obtained

42 $$ \dot{s}\left( t \right) = \dot{u}_{e} + \beta u_{e} $$

It is desired that the controller can make nonlinear system reach the sliding surface *s*(*t*) = 0. By using sliding-mode surface function, the trajectory tracking controller is derived.

#### **Theorem 2**

*Considering posture tracking error* (30) *and dynamics Eq.* (8)*. If we design controller*

43 $$ \begin{aligned} \tau & = \bar{B}^{ - 1} \bar{M}\dot{u}_{c} - \beta \bar{B}^{ - 1} \bar{M}u_{e} \\ & \quad + \bar{B}^{ - 1} \bar{V}u - \bar{B}^{ - 1} \bar{M}\left[ {\rho_{1} \tanh (k_{5} s) + \rho_{2} s} \right] \\ \end{aligned} $$

*the closed*-*loop system is asymptotically stable, where ρ*_*1*_*, ρ*_*2*_*and k*_*5*_*are positive constants.*

#### *Proof*

The dynamics Lyapunov function can be defined as

44 $$ V_{2} = \frac{1}{2}ss^{T} $$

Substituting (41), (42) for differentiation of (44), we obtain

45 $$ \dot{V}_{2} = s^{T} \dot{s} = s^{T} \left( {\dot{u} - \dot{u}_{c} + \beta u_{e} } \right) $$

Substituting (43) for (45), and noting dynamics Eq. (8), we obtain

46 $$ \begin{aligned} \dot{V}_{2} &= s^{T} \left( {\bar{M}^{ - 1} \bar{B}\tau - \bar{M}^{ - 1} \bar{V}u - \dot{u}_{c} + \beta u_{e} } \right) \hfill \\ \, &= - \rho_{1} s^{T} \tanh (k_{5} s) - \rho_{2} s^{T} s \hfill \\ \end{aligned} $$

From (46) it can be concluded that $\dot{V}_{2}$ is the negative semi-definite. It is noted in (41) that $u_{e} = - \beta \int_{0}^{t} {u_{e} } dt$ if the system is on the sliding surface *s*(*t*) = 0. It is obvious that the tracking error lim_*t*→∞_*u*_*e*_ = 0 as *β* > 0. By dynamics controller (43), the system is asymptotically stable. This completes the proof of the theorem. $\hfill\square$

## Simulation results

### Simulation of trajectory planning

In order to verify the effectiveness of the trajectory planning, simulation experiments are performed. The simulation environment is based on Matlab. Assuming that the parameters of the vehicle are set as follows: the curvature radius of the outside lane $R_{2} = 121\;{\text{m}}$ and the curvature radius of the inside lane $R_{1} = 100\;{\text{m}}$, the beginning time $t_{0} = 0\;{\text{s}}$ and the finishing time $T = 18\;{\text{s}}$, the curvature radius of trajectory arcs $\rho = 60\;{\text{m}}$, $\alpha = 0.7\;{\text{rad}}$. The initial state values of vehicle are shown in Table 1.

**Table 1:** Initial state of vehicle

State	Description	Value
*v*_*x*_(*t*_0_)	Longitudinal velocity at time *t*_0_	$5\;{\text{m/s}}$
*v*_*y*_(*t*_0_)	Lateral velocity at time *t*_0_	$0\;{\text{m/s}}$
*a*_*x*_(*t*_0_)	Longitudinal acceleration at time *t*_0_	$0\;{\text{m/s}}^{2}$
*a*_*y*_(*t*_0_)	Lateral acceleration at time *t*_0_	$0.4\;{\text{m/s}}^{2}$
*v*_*x*_(*T*)	Longitudinal velocity at time *T*	$3.6\;{\text{m/s}}$
*v*_*y*_(*T*)	Lateral velocity at time *T*	$0.6\;{\text{m/s}}$
*a*_*x*_(*T*)	Longitudinal acceleration at time *T*	$0.09\;{\text{m/s}}^{2}$
*a*_*y*_(*T*)	Lateral acceleration at time *T*	$0\;{\text{m/s}}^{2}$

**Fig. 6:**
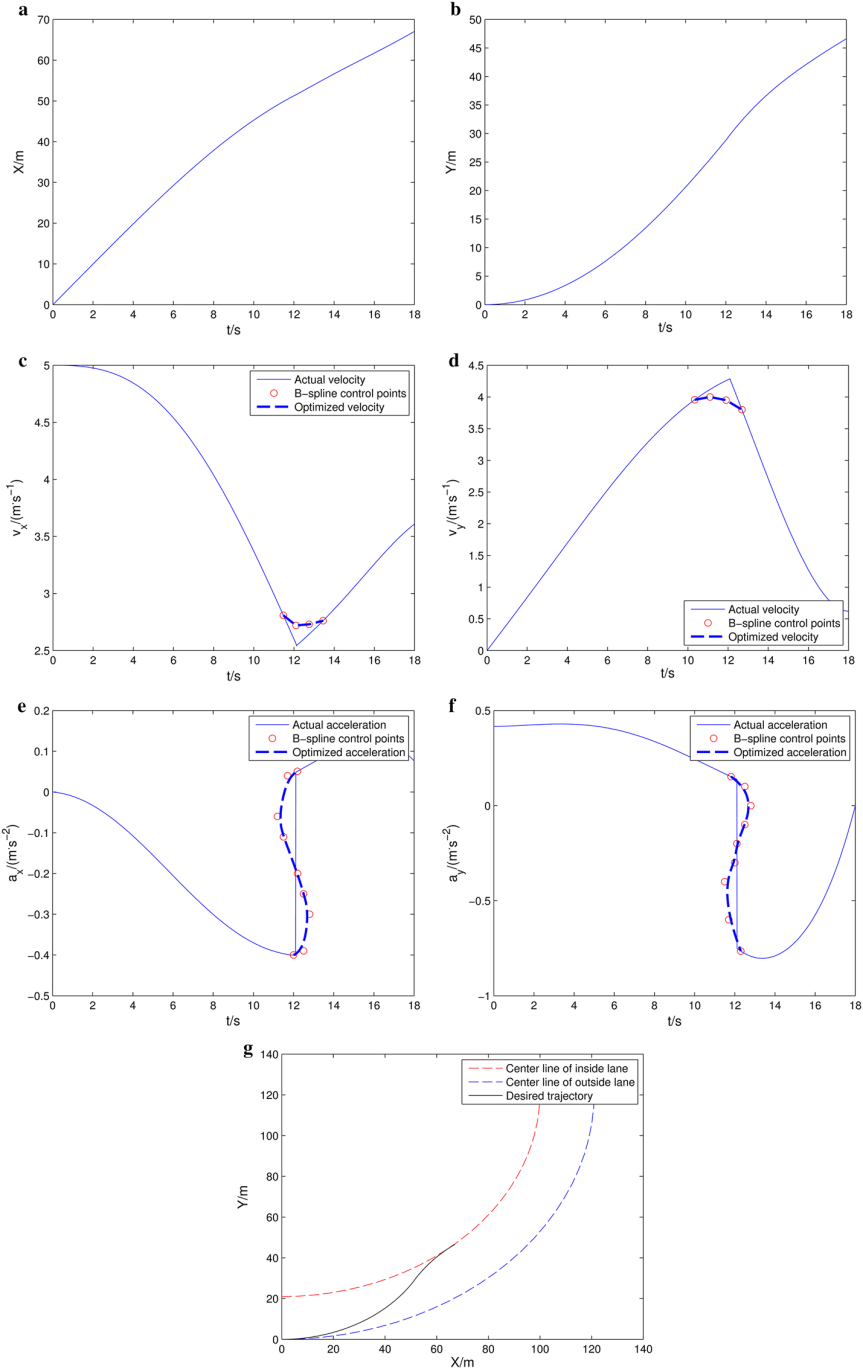
Desired trajectory, velocity and acceleration. **a** X-axis displacement, **b** Y-axis displacement, **c** X-axis velocity, **d** Y-axis velocity, **e** X-axis acceleration, **f** Y-axis acceleration, **g** desired trajectory

The desired trajectory, velocity and acceleration curves are shown in Fig. 6. Figure 6a and b are the displacement along X-axis and Y-axis, Fig. 6c and d are the velocity curve along X-axis and Y-axis and Fig. 6e, f is the acceleration curve.

The solid line in the Fig. 6c–f is the actual velocity and acceleration curve and the dotted line is the optimized velocity and acceleration curve using for B-spline. The red ‘O’ represents the B-spline control points. The actual velocity and acceleration curve have abrupt curvature change in the process of lane changing. It does not meet the LCCP *constraint 1*. We adopt the B-spline control theory to create a smoothness curve. In 2003, Kano et al. put forward the B-spline theory which can be used for curve-fitting and numerical differentiation. The piecewise continuous B-spline function can be defined as

47 $$ Q_{k,n} \left( u \right) = \sum\limits_{i = 0}^{n} {P_{i + k} \cdot F_{k,n} \left( u \right)} $$

where *k* = 0, 1, …, *m*, *P*_*j*_, *j* = 0, 1, …, *n* + *m* are the control points, *u* ∊ [0, 1], *F*_*k*,*n*_(*u*)is the basis function. The B-spline function (47) means the *k* piece *n*-degree B-spline curve.

Noting that the desired trajectory state Eqs. (12)–(14) and (23)–(25) are third-degree derivable, the cubic spline function can be set as the basis function which can be represented by the form of matrix

48 $$ F_{k,3} \left( u \right) = \frac{1}{6}\left[ {\begin{array}{*{20}c} {u^{3} } & {u^{2} } & u & 1 \\ \end{array} } \right]\left[ {\begin{array}{*{20}c} { - 1} & 3 & { - 3} & 1 \\ 3 & { - 6} & 3 & 0 \\ { - 3} & 0 & 3 & 0 \\ 1 & 4 & 1 & 0 \\ \end{array} } \right] $$

According to (47) and (48), the B-spine optimization curve can be calculate as

49 $$ \left[ {\begin{array}{*{20}c} x & y \\ \end{array} } \right] = \frac{1}{6}\left[ {\begin{array}{*{20}c} {u^{3} } & {u^{2} } & u & 1 \\ \end{array} } \right]\left[ {\begin{array}{*{20}c} { - 1} & 3 & { - 3} & 1 \\ 3 & { - 6} & 3 & 0 \\ { - 3} & 0 & 3 & 0 \\ 1 & 4 & 1 & 0 \\ \end{array} } \right]\left[ {\begin{array}{*{20}c} {P_{0,i,x} } & {P_{0,i,y} } \\ {P_{1,i,x} } & {P_{1,i,y} } \\ {P_{2,i,x} } & {P_{2,i,y} } \\ {P_{3,i,x} } & {P_{3,i,y} } \\ \end{array} } \right] $$

where *P*_0,*i*,*x*_… *P*_3,*i*,*x*_are the horizontal coordinates of the B-spline control points, *P*_0,*i*,*y*_…*P*_3,*i*,*y*_ are the vertical coordinates of the B-spline control points. After optimization of the spline function, the velocity and acceleration curve can avoid the occurrence of the abrupt curvature change which meets the requirements of the intelligent vehicle dynamics.

The lane changing trajectory is shown in Fig. 6g. The dotted line is the center line of the two lanes and the solid line is the actual lane changing trajectory.

### Simulation of trajectory tracking

In this section, lane changing trajectory is applied to the reference trajectory which is designed in “Trajectory planning” section. Simulation is designed to show the effectiveness of designed kinematic controller (32) and dynamics controller (43).

The control parameters of the vehicle are set as: *k*_1_ = 2, *k*_2_ = 2, *k*_3_ = 30, *k*_4_ = 30, *k*_5_ = 1, *ρ*_1_ = 10, *ρ*_2_ = 20, *λ* = 0.05, *β* = 1. The physical parameters of vehicle are set as Table 2.

**Table 2:** Physical parameters of vehicle

Parameters	Description	Value
*m*	Mass of vehicle	$1900\;{\text{kg}}$
*I*_*z*_	Moment of inertia	$3 9 0 0\;{\text{kg}} \times {\text{m}}^{2}$
*r*	Radius of the wheels	$30\;{\text{m}}$
*b*	The half of distance between the two wheels	$1. 3\;{\text{m}}$

**Fig. 7:**
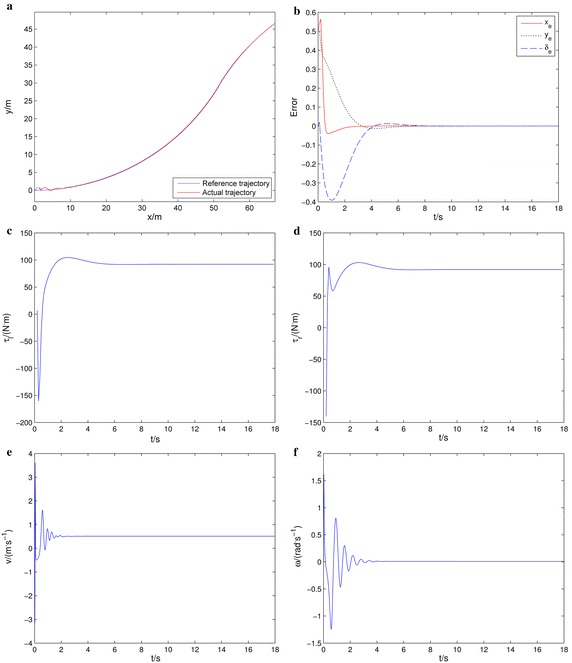
Trajectory tracking. **a** Actual and reference trajectories, **b** tracking errors, **c** torque control input of left wheels, **d** torque control input of right wheels, **e** linear velocity, **f** angular velocity

Simulation results are shown in Fig. 7. Figure 7a describes the actual and reference trajectory. The two trajectories are almost coincident by means of the kinematic and dynamics controller. The tracking errors are shown in Fig. 7b which converges to zero asymptotically. The torque control input of the left and right wheels are shown in Fig. 7c and d. The torque control input converges to a stable range. The linear velocity and angular velocity are shown in Fig. 7e, f. From these results, we can confirm the effectiveness of the proposed method.

## Conclusion

This paper considers lane changing trajectory planning and tracking control for intelligent vehicle on curved road. With the consideration of vehicle dynamics constraints, LCCP constraints are proposed to validate the effectiveness of desired trajectory. Then the arc trajectory with the same curvature is designed to implement lane changing on curved road. Kinematic controller based on backstepping method and sliding-mode dynamics controller are designed to implement the trajectory tracking control. The stability of the control system is proved by Lyapunov stability theory. Simulation results show that the controller can guarantee the convergence of the tracking error.
